# Directed evolution of *Metarhizium* fungus improves its biocontrol efficacy against *Varroa* mites in honey bee colonies

**DOI:** 10.1038/s41598-021-89811-2

**Published:** 2021-05-19

**Authors:** Jennifer O. Han, Nicholas L. Naeger, Brandon K. Hopkins, David Sumerlin, Paul E. Stamets, Lori M. Carris, Walter S. Sheppard

**Affiliations:** 1 Department of Entomology, Washington State University, Pullman, WA USA; 2 Fungi Perfecti LLC, Olympia, WA USA; 3 Department of Plant Pathology, Washington State University, Pullman, WA USA

**Keywords:** Evolution, Microbiology

## Abstract

Entomopathogenic fungi show great promise as pesticides in terms of their relatively high target specificity, low non-target toxicity, and low residual effects in agricultural fields and the environment. However, they also frequently have characteristics that limit their use, especially concerning tolerances to temperature, ultraviolet radiation, or other abiotic factors. The devastating ectoparasite of honey bees, *Varroa destructor,* is susceptible to entomopathogenic fungi, but the relatively warm temperatures inside honey bee hives have prevented these fungi from becoming effective control measures. Using a combination of traditional selection and directed evolution techniques developed for this system, new strains of *Metarhizium brunneum* were created that survived, germinated, and grew better at bee hive temperatures (35 °C). Field tests with full-sized honey bee colonies confirmed that the new strain JH1078 is more virulent against *Varroa* mites and controls the pest comparable to current treatments. These results indicate that entomopathogenic fungi are evolutionarily labile and capable of playing a larger role in modern pest management practices.

## Introduction

Biological pesticides based on naturally occurring microbes that infect pest species have been available to growers in the U.S. and global markets for decades, but they have failed to find widespread use^1^. These microbial biopesticides are considered to have inherently reduced risk by the U.S Environmental Protection Agency due to a suite of favorable toxicological characteristics^2^. Compared to traditional chemical synthetic pesticides, microbial biopesticides generally have very low toxicity to humans and other vertebrates, fast decomposition leading to reduced residues and environmental pollution, and high species specificity leading to reduced non-target effects^2^. Additionally, microbial biopesticides are easily integrated into the growing market for certified organic food^3^, and pests may be slower to evolve resistance to microbial biopesticides than to traditional chemical pesticides^4^.

Although biopesticides make up an increasing percentage of global pesticide usage, they have generally failed to supplant traditional synthetic chemical pesticides outside of certain niche markets^5^. One of the primary reasons is that the effectiveness of microbes for pest control is frequently limited by the susceptibility of the microbes to temperature, ultraviolet radiation, pH, or other abiotic factors^6–8^. This susceptibility to environmental stress shortens the duration of treatment and lowers the overall pest control gained from each application. Increasing the tolerance of microbial biopesticides to abiotic factors is acknowledged as an elusive yet key transformation step if they are to find wider use^1,7^.

*Varroa destructor* is an ectoparasite of honey bees widely considered to be the primary driver of declining honey bee health in recent decades. Although a variety of interacting factors including pathogens, pesticides, and nutritional stress have contributed to declining honey bee health^9–12^, the *Varroa* mite is the most commonly reported cause of colony loss for commercial beekeepers in the United States^13,14^ and is considered the single greatest threat to apiculture world-wide^15^. This, in turn, threatens the ~ $238 billion in crops worldwide that require insect pollination^16^. *Varroa* feed on adult and immature honey bees by puncturing the exoskeleton with sharp mouthparts and consuming bee tissues through extra-oral digestion^17^. Feeding by *Varroa* weakens bees, reduces worker life-span and foraging capability, and vectors some of the most destructive honey bee viruses including Deformed Wing Virus, Israeli Acute Paralysis Virus, Kashmir Bee Virus, and Sacbrood Virus^18,19^. If left untreated, *Varroa* infected colonies have an expected lifespan of 1–3 years^15,19^. Additionally, *Varroa* infected bees are more likely to drift to neighboring colonies, introducing *Varroa* and associated viruses to uninfected colonies^20,21^. This is especially problematic for bees in commercial pollination settings where thousands of hives from different beekeepers and locations are crowded seasonally into orchards and agricultural fields^22,23^.

Currently, beekeepers are largely reliant on chemical acaricides to control *Varroa* despite the dangers that these chemicals pose to bees and the ongoing issues with chemical resistance in the mites. These acaricides have been linked to numerous honey bee health problems, and many studies have shown that residues can accumulate in the hive over time^24–26^. Unintended effects on honey bees include increased mortality in brood and adults^27^ and an increased susceptibility to pathogens and agrichemicals^28–30^. There is a growing body of evidence that links acaricides to reproductive issues in both queens^31–33^ and drones^34,35^. Additionally, interactions between different chemical acaricides can increase their toxicity to bees^36,37^ and breakdown metabolites have been shown to have toxic effects as well^38,39^. Compounding the situation further are the many other insecticides, fungicides, and other agrichemicals that honey bees encounter while foraging in and around agricultural fields^40^. Traditional chemical acaricides also present logistical issues to beekeepers including increased personal protection equipment requirements and prohibition of treatment during honey production. *Varroa* have repeatedly evolved resistance to the chemical acaricides most commonly used by beekeepers, including multiple pyrethroids^41^, the organophosphate coumaphos^42^, and the amidine amitraz^43^.

Several laboratories have demonstrated that *Varroa* are susceptible to entomopathogenic fungi, including *Beauvaria bassiana*, *Hirsutella thompsonii*, and *Metarhizium anisopliae*^44–47^. Field tests with *Metarhizium* showed the fungus was capable of controlling mites^47,48^, sometimes with results comparable to the commonly used chemical acaricides of the time^49,50^. However, despite attempts with several different formulations, commercially available entomopathogenic fungi that were tested generally suffered from low consistency in their ability to control *Varroa*^51^. Researchers repeatedly noted that the relatively warm temperatures found in honey bee hives, 35 °C^52^, was detrimental to the survival and infection potential of spores, leading to rapid decreases in treatment efficacy^43,53^. This situation is compounded by the life cycle of *Varroa,* which spend much of their life living inside closed brood cells with developing bee pupae. This shelters the mites from treatments with short persistence, allowing mite levels to quickly reestablish^51,54^.

Although researchers have now screened and tested dozens of existing strains of entomopathogenic fungi for their potential for *Varroa* control, no one has yet attempted to create a strain specifically for *Varroa* control through any means of genetic manipulation or repetitive selection. In addition to showing promise as a microbial biopesticide against *Varroa*, the *Metarhizium* PARB clade (including the species *M. anisopliae* and *M. brunneum*), has shown itself to be modifiable through genetic engineering^55^ or mutagenesis sectorization screening^56^. In the following experiments, we subjected a strain of *Metarhizium brunneum* to repeated cycles of selection using both directed evolution in laboratory incubators and repetitive selection in full-sized honey bee colonies. This resulted in strains that are better able to survive under bee hive conditions and are better able to control *Varroa* than parental strains.

## Results and discussion

Initial trials of *Metarhizium* for *Varroa* control used 30 established full-sized honey bee colonies that were divided into three groups, balancing each group for colony population and starting mite levels. The F52 strain of *Metarhizium brunneum* (ATCC #90448) was chosen for testing because of its reported efficacy against *Varroa*^47–49^, its genetic manipulability^55^, and evidence that the pathogenicity and control potential of current strains can be improved^57^. In addition, we tested a related strain of *Metarhizium brunneum*^56^ that displays delayed spore production to test for behavioral or pest control differences of *Metarhizium* hyphae as compared to spores. Control colonies received uninoculated agar. The treatment of hives consisted of inverting agar discs from a 95 mm plastic petri dish onto the top bars of the frames of comb in the hive, one disc per box.

**Figure 1:**
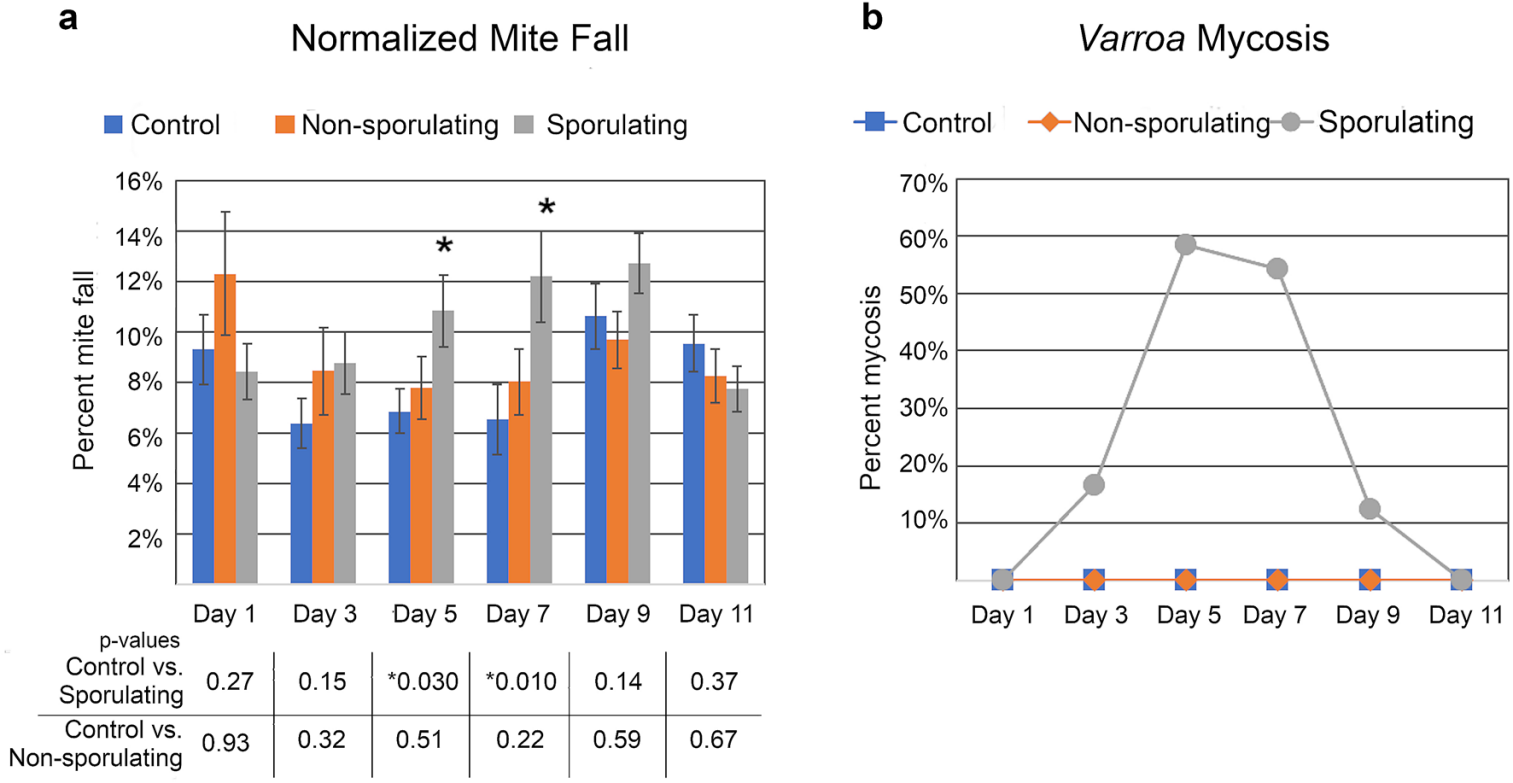
The effects of *Metarhizium* treatment on *Varroa* mite levels. (**a**) Mite fall onto sticky cards in hives treated with pre-sporulating or sporulating strains of *Metarhizium*. Data are normalized to total number of fallen mites for each colony. Number of colonies = 10 (control), 10 (non-sporulating), 10 (sporulating). (**b**) Percentage of mites killed by *Metarhizium*. Fallen mites were surface sterilized and plated onto nutrient agar. *Varroa* growing *Metarhizium* were considered to have died from mycosis. N = 84 (control), 156 (non-sporulating), 168 (sporulating). T tests were used to determine significance.

Treatment with *M. brunneum* F52 that was producing mitospores (asexual spores, sometimes referred to as conidia in *Metarhizium*) significantly increased the number of dead mites collected off bottom board sticky cards compared to hives that received uninoculated agar plates (Fig. 1a; day 5 and 7 p < 0.03). *Varroa* control by this sporulating strain, estimated to have delivered 8.76 × 10^8^ mitospores per treatment, peaked between days 5–7 after treatment, corresponding to peak mycosis (Fig. 1b), and then declined back to non-significant levels from day 9 onward. The rapid loss of mitospore viability in this first trial was somewhat expected, as previous researchers have noted the susceptibility of *Metarhizium* to temperatures found in honey bee hives. Worker bees were observed cleaning living fungus off the agar disk promptly after treatment, suggesting that mitospore production by the treatment declined rapidly as the living fungus was removed. However, this action may have facilitated the spread of mitospores from the dish onto living bees and around the colony.

*Metarhizium* that was not producing mitospores did not show any effect of treatment (p > 0.2 for all days) (Fig. 1). Although *Metarhizium* produces destruxins and other compounds with pesticidal properties, these results confirm previous research^51^ showing that mitospores are the necessary infectious agent of *Metarhizium* for *Varroa* control. Although *Metarhizium* hyphae can display some mycoattractant effects^56^, we did not observe any attraction by *Varroa* to the fungus. Therefore, the primary mode of action for *Varroa* control is highly likely through mitospore adhesion and germination on the mite exoskeleton, followed by hyphal penetration through the exoskeleton and proliferation throughout internal tissues of the mite.

**Figure 2:**
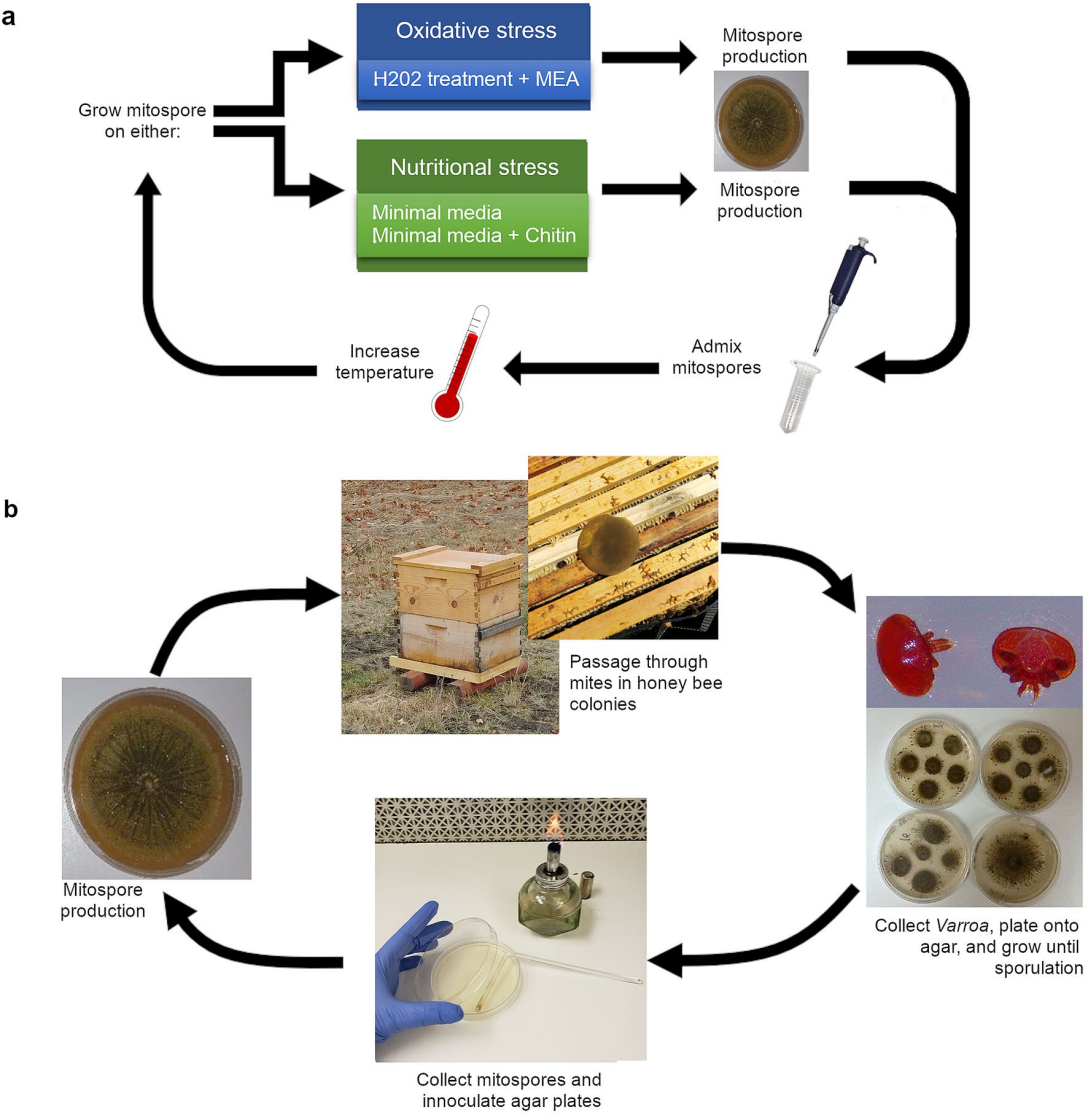
Visual representation of the *Metarhizium* strain creation process. (**a**) In vitro workflow for increasing thermotolerance with directed evolution in *Metarhizium*. (**b**) Workflow procedure for field selection after directed evolution in the laboratory. Mitospores from mycosed *Varroa* cadavers are used to create the next generation of treatment.

Mites from this initial field trial were collected off sticky cards, surface sterilized, and plated on agar. *Metarhizium* that grew out of infected mites were subcultured and used as the starting population for a directed evolution process we designed to induce thermotolerance. The fungus was subjected to repetitive cycles of growth and reproduction under stressful conditions at increasing temperatures (Fig. 2a). The stressful conditions were either oxidative stress and mild mutagenicity induced by hydrogen peroxide treatments or nutritional stress induced by growth on minimal media agar amended with or without chitin. Spores exposed to nutritional stress are better able to withstand UV-stress and heat stress and exhibit increased infectivity^7,58^. There is, however, a tradeoff for fungi grown in nutritionally deficient media; hyphal development is slowed, and mitospore production is decreased^59^. With each repeated cycle the mitospore population was admixed, and the incubator temperature was gradually increased from the ideal growth temperatures for the starting F52 strain (27 °C) to the temperature found in honey bee hives (35 °C).

**Figure 3:**
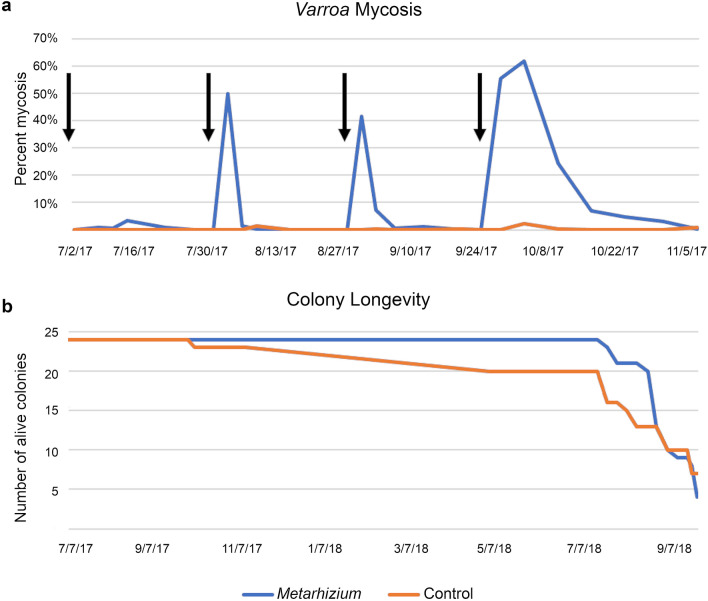
Effects of *Metarhizium* treatment on honey bee colonies. (**a**) Percentage of *Varroa* mites dying from *Metarhizium* mycosis. Black arrows indicate the treatment dates. Following each treatment, *Metarhizium* was recultured from dead mites and used to create the next generation of treatment. Colony N = 24, 24 (treatment, control). (**b**) Longevity of hives in the Stationary Apiary. *Metarhizium* treated hives exhibited longer life span compared to controls (p = 0.022). All hives after August 2018 experienced extreme predation from yellow jackets and eventually perished.

The last generation of spores resulting from the directed evolution process was then used as the starting population for repetitive rounds of field selection. The mitospores were germinated on malt extract agar (MEA) plates, allowed to grow and to produce another generation of mitospores, and then the agar disc was inverted onto the top bars of the frames of comb in full-sized outdoor honey bee colonies (Fig. 2b). A new apiary, designated as the stationary apiary, was established using full-sized colonies started from “two-pound packages” (0.91 kg of bees taken from a common population), with a total of 48 colonies being allocated for repeated treatment with either *Metarhizium* or uninoculated agar discs as controls. The first round of treatment after the directed evolution procedure did not result in high levels of infection in the mites; we were able to reculture living *Metarhizium* from 3.38% of mites collected off of sticky cards (Fig. 3a), indicating that a low number of mites were killed by the fungus. This low number was not unexpected, as many of the genetic changes acquired during the directed evolution process would not be favorable for virulence in living hosts under field conditions. Additionally, repeated subculturing on artificial media is known to decrease virulence in as little as 20 subcultures^60^. Living fungus that was recultured from the infected mites was then grown to sporulation, and the subsequent generation was used to treat the same population of hives again (Fig. 3a). After a single generation of selection through *Varroa* hosts, this treatment resulted in 49.9% of mites dying from mycosis. The process of harvesting mitospores from dead mites, growing another generation, and treating the colony again was repeated two additional times that field season. The final treatment exhibited extended efficacy, lasting up to 5 weeks post treatment (Fig. 3a), indicating increased tolerance to bee hive conditions. No negative effects were detected and the colonies in the treatment and control groups went into winter with similar bee population estimates (t test p = 0.72 see Supplementary Fig. S1 online).

The colonies in the stationary apiary continued to receive treatment of either *Metarhizium* or uninoculated agar the subsequent year, using the same protocol as before. Hives treated with *Metarhizium* survived significantly longer than untreated hives (Fig. 3b; p < 0.02), although 42 of the 48 hives succumbed to *Varroa*, pathogen pressure, and intense yellow jacket predation by the end of year two. *Metarhizium* treatment delayed the exponential increase in *Varroa* levels but did not total prevent it (see Supplementary Fig. S2 online). The presence of untreated colonies in the apiary created what are known colloquially as “mite bombs”^61^. Colonies with high *Varroa* infestation levels continuously inoculate colonies in the area with mites and associated viruses through drifting bees and honey robbing, leading to health problems for all colonies in the apiary. The spreading of mites from untreated colonies to all colonies can be seen in measurements of mite levels from the stationary apiary (see Supplementary Fig. S3 online).

**Figure 4:**
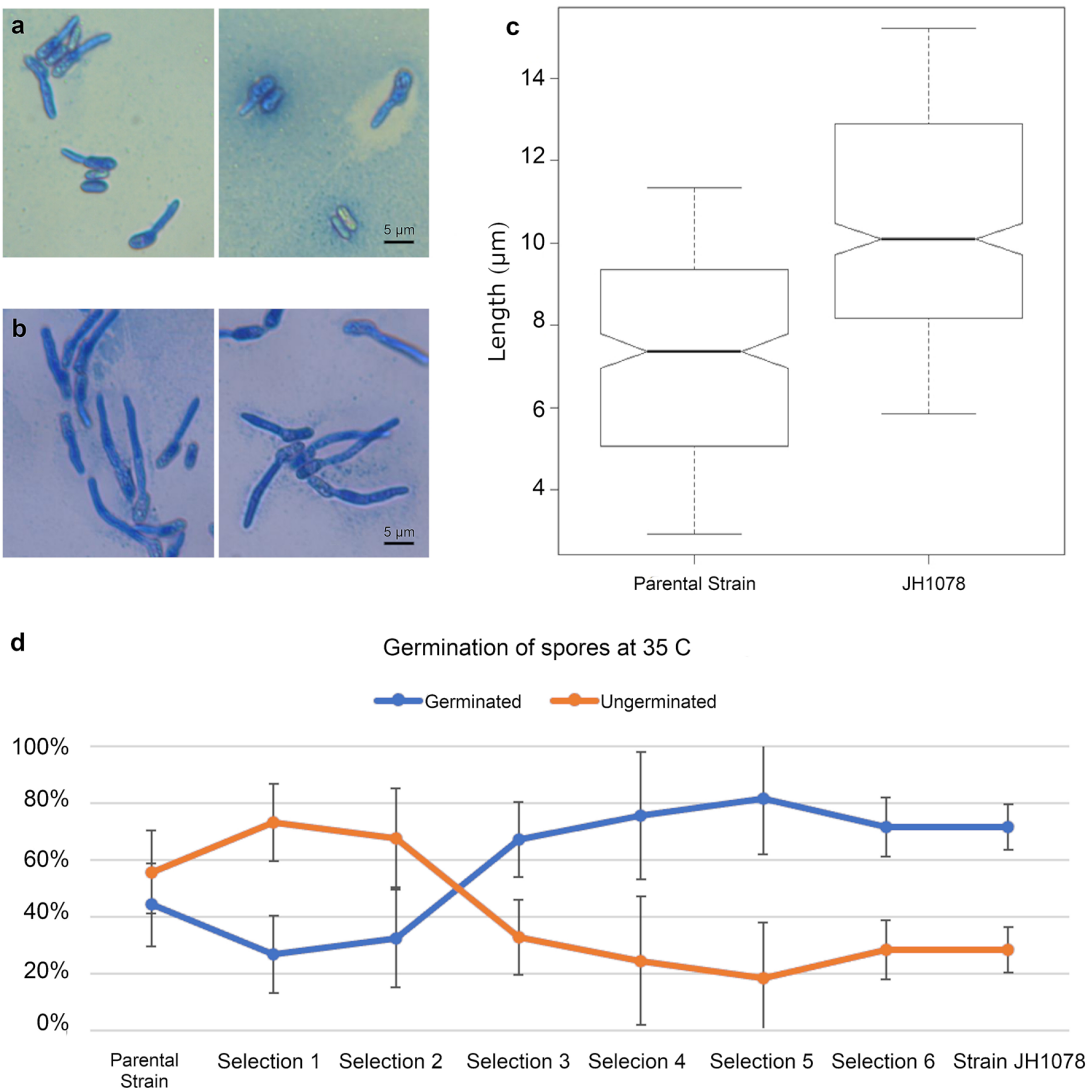
Parental and new strains of *Metarhizium* have distinct phenotypes at 35 °C. (**a**,**b**) Typical germinated mitospores stained with lactophenol cotton blue 24 h after plating and incubation. (**a**) Parental strain, F52; (**b**) strain JH1078; (**c**) average length of germination tube after 24 h of incubation (t test p = 8.5 × 10^–27^). (**d**) Germination success rate of mitospores at 35 °C.

A strain resulting from the selection process at the end of the stationary apiary experiments, designated as strain JH1078, was compared against the parental strain for growth and germination characteristics at 35 °C in a laboratory incubator. There are several significant differences in morphology and longevity between the parental strain F52 and strain JH1078 (Fig. 4a,b). After 24 h incubation at 35 °C, only 44% of the parental strain mitospores were able to germinate, whereas over 70% of strain JH1078 mitospores germinated at 35 °C (Fig. 4d). Of the germinated spores, the parental strain germination tube was on average 4.8 ± 0.20 µm long. Strain JH1078 germination tube was significantly longer (t test p = 8.5 × 10^–27^), on average 10.48 ± 0.17 µm (Fig. 4c).

To test *Metarhizium* JH1078 in the context of modern beekeeping operations, full-sized hives that had participated in migratory commercial pollination from February to June were treated with *Metarhizium* or a common EPA approved *Varroa* control treatment, a 2.8% oxalic acid drip treatment^62^. For these experiments, the JH1078 strain was grown on brown rice and placed into natural fiber mesh bags. Each hive was treated with 120 g of colonized grain bearing 2.63 × 10^8^ spores per gram. Both the *Metarhizium* and oxalic acid treatments were applied twice, 7 days apart. Colonies were sampled for *Varroa* levels using ethanol washes before the treatments and at the end of the experiment 18 days later. In this field relevant situation, *Metarhizium* treated colonies did not differ significantly from the oxalic acid treating colonies and trended towards controlling mites better (Kruskall–Wallis p = 0.33; See Supplementary Fig. S4 online).

## Conclusions

These results reveal the plasticity of *Metarhizium* as a biocontrol agent and demonstrate that novel beneficial phenotypes can be created and selected for with a combination of directed evolution in the laboratory and field selection. Importantly, in an era of declining honey bee health, the strains of *Metarhizium* created in these experiments were able to control *Varroa* mites and may provide beekeepers with an alternative to chemical acaricides. Additionally, it is possible that the methods presented here could be applied to fungi or other biocontrol agents targeting other arthropod pests. Susceptibility to environmental stressors like heat is frequently noted as a factor limiting the efficacy of entomopathogenic fungi, but these results indicate that such barriers can be overcome.

## Materials and methods

### Fungal isolates

The starting strain of *Metarhizium brunneum* that acted as the parental strain before selection was obtained from the American Type Culture Collection (ATCC #90448). The pre-sporulating strain isolate (US Patent #8501207B2) was obtained from Fungi Perfecti LLC. (Olympia, Washington, USA). The strains were stored in the dark on Malt Extract Agar (MEA) at 4 °C. With the exception of growth during the directed evolution experiments using incubators, *Metarhizium* for hive treatment was grown on 95 mm MEA Petri plates at room temperature (25 °C) until the plate was fully colonized and producing mitospores, usually after 12–16 days.

### Initial field trials of starting strains

In August of 2016, fully established honey bee colonies from four different apiaries in Eastern Washington were evaluated for *Varroa* mite levels, amount of brood, and total hive population. Thirty colonies were selected for the experiment and assigned to one of three groups, balancing the groups for these health-related traits. The hives were then moved to a new apiary in Troy, Idaho and installed with raised bottom boards that could accommodate sticky cards. In September, the colonies were treated with either sporulating *Metarhizium*, pre-sporulating *Metarhizium*, or uninoculated MEA substrate. At the end of the trial, hives were treated with a single strip per brood box of CheckMite+ (Bayer Animal Health, USA; active ingredient is the organophosphate Coumophos) to kill the remaining mites and provide an estimate of overall level of mites remaining in the hives. The data presented in Fig. 1a were compiled by normalizing the number of mites caught on the sticky cards over time to the total number of mites that fell throughout the experiment, including in the 1 week after CheckMite+ treatment. Significance was analyzed using t tests (Microsoft Excel).

### In vitro thermotolerance selection

Mitospores were either treated with H_2_O_2_ or grown on minimal media (Czapek Dox Agar without sucrose: NaNO_3_ 0.2%, K_2_HPO_4_ 0.1%, MgSO_4_ 0.05%, KCl 0.05%, FeSO_4_ 0.001%, Bacto Agar 1.5%) with or without chitin (4 g chitin/1 L). Spores treated with H_2_O_2_ were submerged in a 0.3% or 0.03% H_2_O_2_ solution for 60 min. The solution was prepared fresh before use and spores were covered during the treatment to prevent light degradation of H_2_O_2_. After 60 min, the spores were rinsed three times with sterile water before plating on MEA. Agar plates (95 mm; 10 for each treatment) were inoculated with 1 × 10^5^ mitospores mL^−1^ and allowed to grow to sporulation. Plates were incubated at successively increasing temperatures, starting at 27 °C and rising to 35 °C in one degree increments over eight generations.

### Field trials

#### Stationary apiary establishment and maintenance

A stationary apiary consisting of 48 full-sized colonies was established in Moscow, Idaho in April 2017 for long-term field treatment and selection experiments. To limit contact with pesticides and diseased bees from other operations, these hives did not participate in any migratory commercial pollination activity. The field site was within 2 km proximity to urban areas and farms with other small honey bee operations which may have served as sources for mite and pathogen inoculation into our hives. All hives were started using new hive woodware (hive boxes, bottom boards, and lids) and new “two-pound packages” of bees (0.91 kg or ~ 7000 worker bees and a mated queen) from the same commercial bee supplier based in California, USA. Hives were started with two frames of honey, three frames of foundation, four frames of empty drawn comb, and a one-gallon (3.8 L) feeder for sucrose solution feeding during times of low floral abundance.

The hives were placed into four clusters at least 20 m apart with plots of pine trees serving as barriers between clusters. Each cluster contained 12 hives in a horseshoe pattern, with individual hives being spaced 2–3 m apart. Hive entrances were marked with unique color and texture patterns to reduce drift of workers between colonies. The apiary was monitored regularly for 6–8 weeks to ensure all colonies had established and were of approximately similar sizes and health before beginning field tests. During this establishment phase, frames of brood were occasionally moved from strong hives to weak hives to equalize hive populations. Hives were maintained with minimal beekeeper intervention but frequent hive monitoring. Because a swarming event would have considerably affected mite levels, queen cells were removed and honey supers were added as needed. Hives were monitored for brood and frame number to determine what, if any, negative effects treatment might have on colony health.

To prevent colonies that were succumbing to intense *Varroa* infestation from spreading their infections to neighboring hives, hives that were measured to be over 15 mites per 100 bees (five times higher than the treatment threshold) in ethanol samples were removed from the apiary and considered functionally dead for the experiment.

#### Stationary apiary treatment

The colonies in the stationary apiary received either treatment with sporulating *Metarhizium* on MEA agar discs (N = 24) or uninoculated MEA agar discs as a control (N = 24). Hemocytometer counts in the laboratory showed that each *Metarhizium* treatment plate contained on average 8.76 × 10^8^ spores. Hives were treated every 4 weeks over the course of the 2017 and 2018 field seasons. Treatment consisted of inverting Petri dishes (95 mm × 15 mm plates) and removing the agar discs onto the top bars of the honey comb of the hives, one disc for each hive box. Hemocytometer counts in the laboratory showed that each plate contained on average 8.76 × 10^8^ spores.

#### Stationary apiary data collection and analysis

*Varroa* mite population levels in the stationary apiary were assayed twice the first year (July, November) and four times the second year (April, June, August, September) using ethanol sampling of adult bees (after Dietemann^63^, but with increased shaking time to dislodge mites). In addition, bottom board sticky cards were maintained continuously throughout the field season to capture all *Varroa* dying in the hive. The sticky cards were 46 cm × 33 cm cardstock with a thin layer of petroleum jelly (Vaseline) applied to the top side. A screen made from 3.18 mm mesh hardware cloth was placed over the card; bees could walk on the screen, while dead mites and small hive debris could fall through onto the card. Sticky cards were changed every 3 days for the first 15 days after treatment, and then every 7 days until the next treatment. For each card, the number of dead mites were counted, and 12 mites were selected to be analyzed for mycoses. Mites were surface sterilized with 95% ethanol to minimize the possibility of growing *Metarhizium* that was external on mite bodies rather than fungus that had internally infected mites. Surface-sterilized mites were then plated onto 1/4 strength PDA + streptomycin/penicillin antibiotics and incubated at 25 °C until fungal growth was visible. Mites that grew *Metarhizium* colonies after surface sterilization were considered to have died by mycosis. Spores from *Metarhizium* colonies that grew out of mites in these cultures were collected with a dissecting needle and transferred to a 0.01% Tween 80 solution; 15 µL of this solution was spread onto an MEA plate to create the next generation of treatments. Honey bee colony mortality differences were analyzed with OASIS 2^64^. Mantel-Cox tests were used to determine significant differences in mortality between treatments.

#### Mitospore germination/germination tube

*Metarhizium* colonies were grown on potato dextrose agar (PDA). Mitospores were harvested after 2 weeks of growth using a sterile microbiological loop and transferred to a 0.01% Tween 80 solution. Mitospore suspensions (10^5^ mitospores mL^−1^) were shaken using a vortex for 30 s before dropping, without spreading, 30 µL onto 20 mL of 1/4 PDA medium in polystyrene petri dishes (95 mm × 15 mm). Three different mitospore samples were inoculated onto each petri dish. Samples were incubated for 24 h at 35 °C in the dark, an environment that most closely simulates the honey bee hive. Three drops of lactophenol cotton blue stain was added after 24 h, to fix and stain mitospores and prevent further germination. The droplets were covered with a coverslip and examined under a light microscope at 400× magnification. Mitospores were considered germinated if the germ tube was longer than the length of the mitospore^65^. A minimum of 300 mitospores were observed per sample and the percent germination was calculated according to Braga et al.^65^. Germ tube length was measured for a minimum of 300 mitospores per sample using Zen software (Carl Zeiss AG).

#### Metarhizium JH1078 and oxalic acid comparison

In June of 2020, 20 hives in an apiary near Moscow, Idaho were chosen at random to receive *Metarhizium* JH1078 or oxalic acid treatments. *Metarhizium* inoculum was grown on Potato Dextrose Agar (PDA) at room temperature to sporulation (~ 20 days). Organic short grain brown rice (Lundberg Family Farms, Richvale, California) was rinsed and then soaked in 82.2 °C water for 15 min. 3 kg of hydrated grain was then transferred to fungal grow bags (Unicorn Corp.) and sterilized in an autoclave. After cooling, the bags were inoculated with spores from the starting culture by tapping the agar plates upside down over the opening of the bag. The bags were shaken to evenly distribute the spores. Bag cultures were grown to sporulation, and then the sporulating grain was transferred into 7 cm × 10 cm banana fiber bags with 3.18 mm mesh. Oxalic acid treatment was prepared as a 2.8% oxalic acid solution in 1:1 (w/v) sucrose syrup. Five milli litre of this solution was dripped in the gaps between all frames in the hive that were covered with bees. Mite levels were measured with ethanol washes at the start and end of the experiment.

## Supplementary Information

Supplementary Information 1.

## Data Availability

The datasets generated during and/or analysed during the current study are available from the corresponding author on reasonable request. A type specimen of *M. brunneum* JH1078 has been deposited in the United States Department of Agriculture’s Agricultural Research Service Culture Collection (NRRL), accession number NRRL 68016.
