# Supplementary material for: Directed evolution of Metarhizium fungus improves its biocontrol efficacy against Varroa mites in honey bee colonies
Source: Sci Rep. 2021 May 19;11:10582. doi: 10.1038/s41598-021-89811-2 (PMC8134475; doi:10.1038/s41598-021-89811-2)
Supplement: Supplementary file 1 — Supplementary Information 1. [file 41598_2021_89811_MOESM1_ESM.docx]

**Supplementary information**

Supplementary figure S1. Mean frames of bees ± SEM at the end of the year one. Treatment did not affect honey bee frame numbers (t-test p = 0.72).

Supplementary figure S2. Mean *Varroa* population ± SEM of the stationary apiary as determined using ethanol washes. *Metarhizium* treatment delayed the exponential increase in *Varroa* levels but did not totally prevent it.


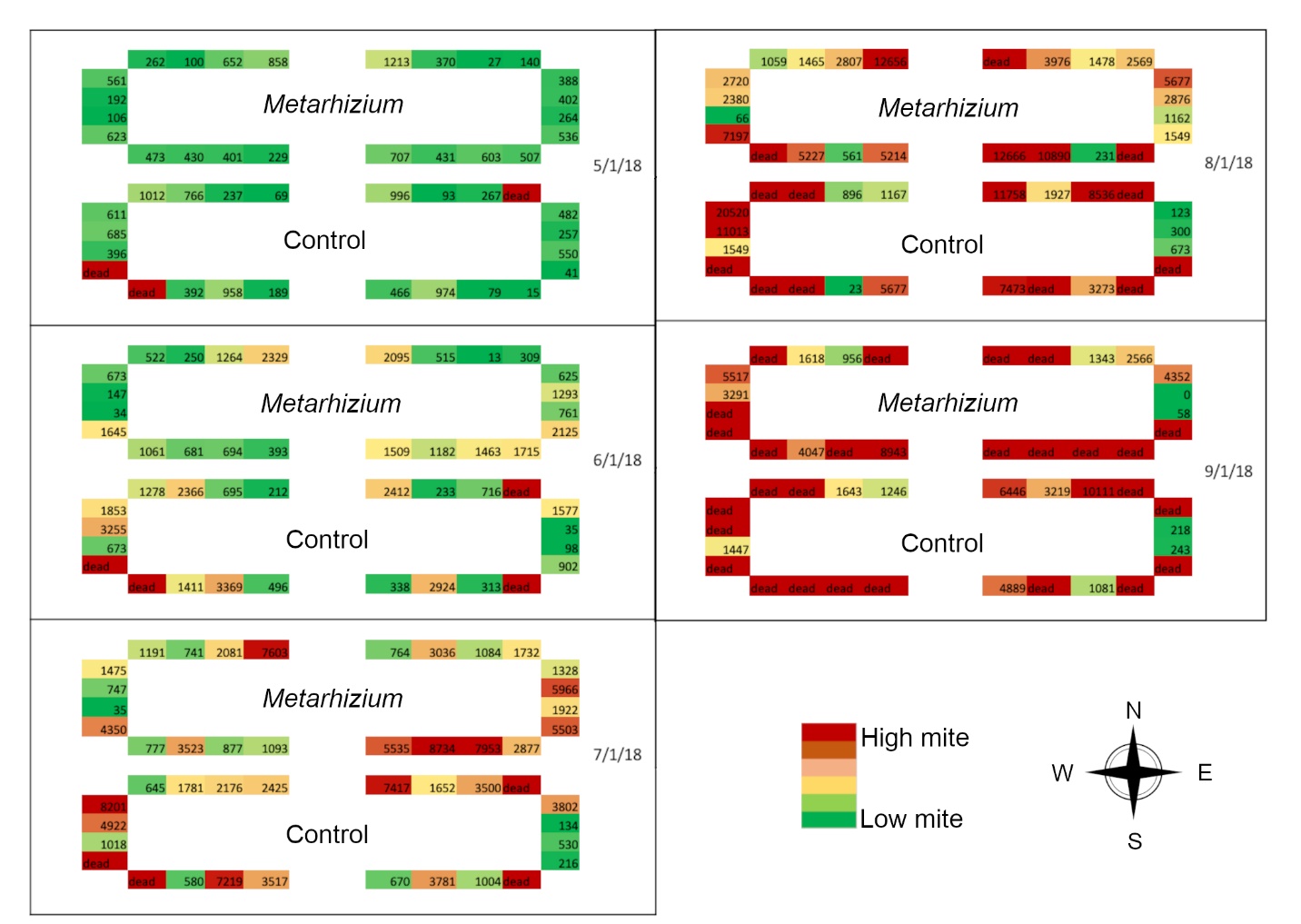


Supplementary figure S3. Total mite drop for each hive in the stationary apiary over one month periods of time throughout the 2018 field season. Data are arranged in accordance with geographical location relative to each other in the apiary.


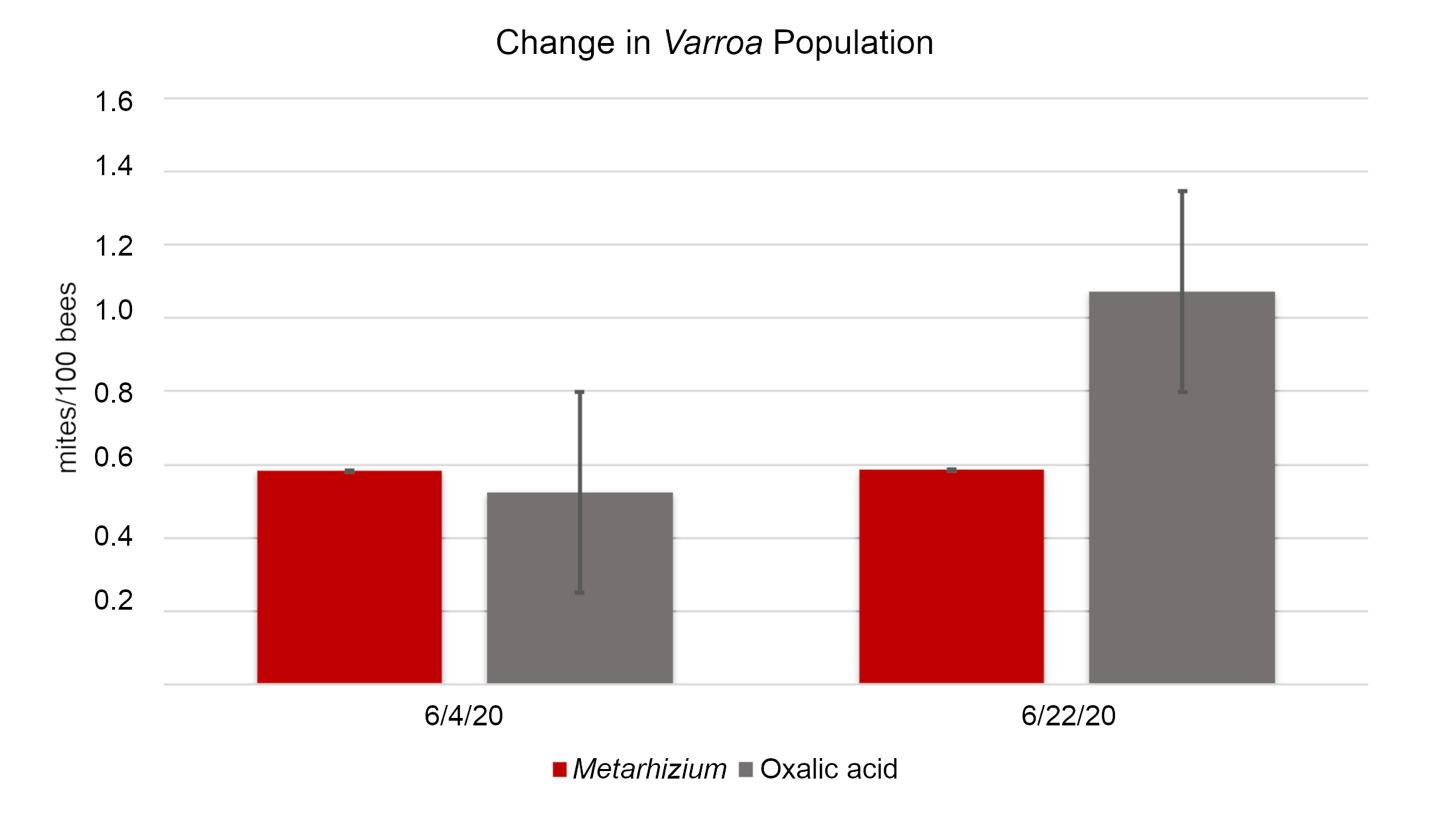


Supplementary figure S4. Mean mite levels ± SEM in colonies treated with *Metarhizium* or oxalic acid, at the start of the experiment and 18 days later. The changes in mite levels were not significantly different between the treatment groups (Kruskall Wallis p=0.33). N=11 (*Metarhizium*, 9 (oxalic acid).
